# Development and Optimisation of Tumour Treating Fields (TTFields) Delivery within 3D Primary Glioma Stem Cell-like Models of Spatial Heterogeneity

**DOI:** 10.3390/cancers16050863

**Published:** 2024-02-21

**Authors:** Callum G. Jones, Aurelie Vanderlinden, Ola Rominiyi, Spencer J. Collis

**Affiliations:** 1 Division of Clinical Medicine, University of Sheffield Medical School, Sheffield S10 2RX, UK; c.g.jones@sheffield.ac.uk (C.G.J.); aurelie.vanderlindendibekeme@pennmedicine.upenn.edu (A.V.); 2 Department of Neurology, Perelman School of Medicine, University of Pennsylvania, Philadelphia, PA 19104, USA; 3 Division of Neuroscience, University of Sheffield Medical School, Sheffield S10 2HQ, UK; 4 Department of Neurosurgery, Sheffield Teaching Hospitals NHS Foundation Trust, Sheffield S10 2JF, UK

**Keywords:** Tumour Treating Fields, TTFields, glioblastoma, glioma stem-like cells, 3D models, DNA damage response inhibitors, standard-of-care therapies

## Abstract

**Simple Summary:**

Glioblastomas are aggressive and therapy-resistant high-grade brain tumours that cause around 200,000 worldwide deaths each year. Tumour Treating Fields (TTFields) therapy represents an important advance in the management of glioblastomas, providing ~five months of survival, which has led to clinical approval in multiple countries worldwide. However, even with this, only around 13% of patients with a newly diagnosed glioblastoma survive more than five years. As such, there is an urgent need to improve our understanding of the cellular responses to TTFields in the context of clinically relevant glioblastoma models and identify new treatment regimens that could augment the effectiveness of TTFields. In this manuscript, we report the development and optimization of a new 3D glioma stem cell model system that facilitates the assessment of TTFields therapies alongside chemoradiotherapy and approved/emerging new therapeutics. This, therefore, provides a key preclinical platform for the development of new TTFields-based approaches to improve the treatment of these currently incurable tumours.

**Abstract:**

Glioblastoma is an aggressive, incurable brain cancer with poor five-year survival rates of around 13% despite multimodal treatment with surgery, DNA-damaging chemoradiotherapy and the recent addition of Tumour Treating Fields (TTFields). As such, there is an urgent need to improve our current understanding of cellular responses to TTFields using more clinically and surgically relevant models, which reflect the profound spatial heterogeneity within glioblastoma, and leverage these biological insights to inform the rational design of more effective therapeutic strategies incorporating TTFields. We have recently reported the use of preclinical TTFields using the inovitro^TM^ system within 2D glioma stem-like cell (GSC) models and demonstrated significant cytotoxicity enhancement when co-applied with a range of therapeutically approved and preclinical DNA damage response inhibitors (DDRi) and chemoradiotherapy. Here we report the development and optimisation of preclinical TTFields delivery within more clinically relevant 3D scaffold-based primary GSC models of spatial heterogeneity, and highlight some initial enhancement of TTFields potency with temozolomide and clinically approved PARP inhibitors (PARPi). These studies, therefore, represent an important platform for further preclinical assessment of TTFields-based therapeutic strategies within clinically relevant 3D GSC models, aimed towards accelerating clinical trial implementation and the ultimate goal of improving the persistently dire survival rates for these patients.

## 1. Introduction

Glioblastomas cause around 200,000 worldwide deaths each year and are the most common, aggressive and deadly central nervous system malignancy [1,2,3,4]. The current standard of care (SoC) consists of maximal safe surgical resection (where safe/appropriate to do so) of the bulk tumour followed by adjuvant chemoradiotherapy to try and eliminate residual disease comprised of brain invasive tumour margin (edge cells) using the DNA methylating agent temozolomide (TMZ) in combination with radiotherapy (RT), followed by cycles of adjuvant TMZ [5,6]. Despite this, relapse within 6–7 months post-surgery is very common due to treatment-resistant repopulating residual disease which contributes to a poor prognosis and median overall survival of 12–15 months [7]. Such dismal statistics are largely attributed to treatment resistance owing to profound spatial, temporal and sub-cellular tumour heterogeneity, the impact of which includes divergent therapeutic responses between cells within the tumour core (including the disease normally resected at surgery) and the invasive margin/edge (residual) tumour cells, which disproportionately give rise to disease recurrence following treatment. This is further exacerbated by the presence of glioma stem-like cell (GSC) sub-niches which are characterised as chemoradiotherapy-resistive due to their heightened DNA damage response (DDR) and high levels of plasticity [8,9,10,11,12,13,14,15,16]. This complex tumour heterogeneity, both within and between patients, highlights the need for tailored and more multifaceted treatment regimens to provide effective, durable disease control for future patients.

Tumour Treating Fields (TTFields) therapy is available as the Optune Gio^TM^ system produced by Novocure and offers a clinically approved fourth modality in glioblastoma treatment alongside chemotherapy in countries such as the USA, Israel, Japan, China and a number of European countries [17]. TTFields are alternating, low-intensity electric fields operating at intermediate frequency, which exert forces on dipolar and charged molecules and can therefore disrupt a range of biological processes within cells. Attuned to a specific frequency optimised to disproportionately impact cancerous cells over healthy tissue (200 kHz in the context of glioblastoma), these disruptive effects are most pronounced during mitosis due to the interference of microtubule polymerisation by dipolar tubulin subunits [17]. These mitotic aberrations can initiate cell death mechanisms through apoptosis, autophagy and immunogenic pathways. TTFields-mediated therapeutic effects have also been implicated in tumour cell toxicity through downregulation of key DDR proteins such as BRCA1/2 and components of the Fanconi anaemia pathway; induction of replication stress; prevention and targeting of metastasis; and invasion and reversible permeabilisation of cancer cell membranes and the blood–brain barrier, leading to improved chemotherapeutic drug delivery (see [17] and the references therein).

As such, hyperplastic and more rapidly dividing cancer cells/GSCs within brain tumour niches are more susceptible to the cytotoxic effects of TTFields than the surrounding normal/differentiated tissue, either alone or alongside chemoradiotherapy strategies [17,18,19]. Indeed, we have recently demonstrated significantly increased TTFields potency towards GSCs when combined with IR and/or a range of clinical DDR inhibitors (DDRi [20]). Therefore, TTFields have the potential to support multiple combinatorial strategies to treat glioblastoma. This therapeutic opportunity highlights the need to develop effective, robust and accurate preclinical in vitro models which are patient-relevant, clinically predictive and have the throughput capacity to evaluate novel TTFields-based treatment strategies for glioblastoma at a sufficient scale to efficiently prioritise these for in vivo studies and future clinical trials [21].

Currently, 2D cell culture monolayers on plasticware are widely used to test TTFields-based multi-modal treatments due to their low cost, ease of setup and alignment with currently available experimental protocols for the inovitro^TM^ TTFields delivery system. However, these models fail to accurately replicate the 3D GSC environment and dynamics of cell-to-cell interaction in vivo, which results in poor clinical predictability including the response to standard of care chemoradiotherapy at the preclinical stage [22,23,24]. Previous studies have highlighted that in vitro Alvetex^TM^ scaffold-based 3D GSC models more accurately reflect therapeutic responses and patient outcomes in the clinic [23,24]. However, there are currently no reports on, or established protocols for, the preclinical delivery of TTFields within 3D scaffold-based architectures.

Here we present the first reported development and optimisation of a method to deliver TTFields within 3D scaffolds, underpinned by our recent 2D GSC TTFields studies [20]. We further use these 3D models to assess TTFields together with TMZ and PARPi treatments within parallel, clinically relevant spatial heterogeneity GSC models derived from the tumour core and invasive edge of the same glioblastoma. These proof-of-concept data confirm biologically impactful preclinical TTFields delivery within the 3D scaffolds and, encouragingly, demonstrate significantly improved GSC cell killing from the combination treatments compared with individual treatment irrespective of GSC spatial origin. This methodology therefore provides an important platform on which to test further potential TTFields-based therapeutic strategies within clinically relevant cancer models that can better reflect the complex, heterogeneous niches and phenotypes that represent a current barrier to successful treatment regimens.

## 2. Materials and Methods

### 2.1. Patient Recruitment and Sample Transfer

G1 GSC cells were generated in Cambridge within Professor Colin Watts’s laboratory and have been previously used in reports from multiple groups, including our own [20,25,26]. The primary glioblastoma OX5 core and invasive edge 3D GSC models were generated from fresh treatment-naïve glioblastoma tissue (based on neurosurgeon guidance) collected from consenting patients undergoing surgery at Sheffield’s Royal Hallamshire Hospital (ethical approval: Yorkshire & The Humber—Leeds East REC 11-YH-0319 (STH15598)), as previously described [27,28]. Only glioblastoma tissue surplus to histological requirements was used and it was resected by the operating surgeon, collected intraoperatively whilst surgery was ongoing, pseudonymised/assigned a unique sample identifier and then rapidly transferred to the research laboratory in a dry, sterile specimen pot (NHS) at room temperature within 10 min of collection from surgery.

### 2.2. Deriving and Culturing Primary GSCs

The GSC culture medium consisted of advanced DMEM-reduced serum, 1% B27 and 0.5% N2 supplements, 1% L-glutamine, 1% Penicillin-streptomycin, 1% Amphotericin B, 0.1 mg/mL EGF and 0.1 mg/mL FGF. GSCs were cultured in plasticware coated with growth-factor-reduced Cultrex^®^ basement membrane extract to promote adherence and maintenance of the GSC phenotype. Cultrex^®^ was aliquoted into 1.5 mL Eppendorf tubes and stored at −80 °C until required, whereby thawing was performed overnight by placing the Eppendorfs in ice at 4 °C. The following day, Cultrex^®^ was diluted into cold, unsupplemented advanced DMEM at a 1:40 dilution. Cultrex^®^ solution was added to plasticware T-75 (2.5 mL/flask) and Alvetex^TM^ 12-plates (500 μL/well) and incubated at 37 °C for 30 min prior to use to permit polymerisation, before aspiration of any excess coating solution. All cell lines used were kept at early passage (5–12 sub-passages from initial derivation) and cultured in GSC culture medium throughout to maintain the GSC phenotype and remove any non-cancerous cells, as previously described [27,28].

### 2.3. Delivery of TTFields

The inovitro^TM^ system (NovoCure Ltd.; Haifa, Israel) was used to generate TTFields [29], with GSC cultures seeded and treated as previously described [20] and outlined in more detail within the results section below. Dishes containing seeded GSCs that were to receive TTFields treatment were connected to a generator to produce alternating electric fields at the frequency clinically approved for the treatment of glioblastoma (200 kHz), with the directionality of electric fields treatment applied alternating by 90° every 1 s [29]. As the delivery of the electric fields generates micro-heating within the dish, dependant on the intensity of the applied field, the base plate with connected dishes was placed in a refrigerated incubator (with 5% CO_2_ and 21% O_2_) in order to maintain the temperature of the treated dishes at 37 °C throughout the treatment. The incubator was set at a temperature of 22 °C, equating to a maintained TTFields intensity of 1.33 V/cm root-mean square (RMS) at 37 °C [29]. Cells were treated for a duration of 72 h based on calculated cell doubling times, as described in more detail within the results.

### 2.4. 3D Clonogenic Survival Assays

Following treatment with DDRi, IR and/or TTFields, GSCs were harvested and reseeded in matrigel-coated 6-well tissue culture plates at varying densities (between 500 and 1000 cells/well) and incubated for 21 days. Following the 21-day incubation period, 200 μL of MTT reagent (10 mg/mL in PBS) in 2 mL of GSC media was added to each well and then incubated for 4 h at 37 °C in the dark. Importantly, in 3D clonogenic studies, MTT is used only to provide colony-specific staining (since methylene blue, which is traditionally used to stain colonies, can also discolour the Alvetex^TM^ scaffolds) and the formazan product is not solubilised to estimate cellular activity or viability [23,24]. Subsequently, MTT-containing media was aspirated off the scaffolds, and 200 μL/well of 4% paraformaldehyde was added. Plates were incubated at room temperature for 15 min to allow fixation of the colonies, before removal of the excess paraformaldehyde. Colonies were counted and the plating efficiency (untreated control) was used to calculate the surviving fractions: number of counted colonies/(number of cells plated × PE).

### 2.5. Preparation of Cell Lysates for Downstream Analyses

Cell lysis was performed as previously described [20], but briefly, the lysis buffer (10 mL) consisted of 500 μL 1 M Tris pH 8, 400 μL 5 M NaCl, 100 μL Triton X, 10 μL 1 M DTT, 20 μL 500 mM EDTA, 20 μL 250 units/mL benzonase, 1× Phosphostop tablet, 1× complete EDTA-free protease inhibitor and 8.96 μL ddH2O. The optimised methodology to generate protein extracts from 3D-cultured GSC models is described in detail below. For protein quantification, 1:5 diluted bio-rad protein assay dye concentrate (200 μL in 800 μL ddH_2_O) was added to 3 μL of lysate and the 595 nm absorbance measured on a plate reader. Protein concentration was then determined from a BSA standard curve.

### 2.6. SDS-PAGE

35 μg of protein and 4× NuPage LDS Loading Buffer mix were loaded into each lane of a NuPAGE 4–12% Bis-Tris gradient gel and electrophoresed for ~75 min at 140 V. Proteins were then transferred to nitrocellulose membranes at 100 V for 180 min in Mini PROTEAN Tetra Cells, using 1× NuPAGE transfer buffer (20× stock) diluted with pure methanol and ddH_2_O. Membranes were blocked for 60 min in 5% milk with phosphate-buffered saline (Thermo Fisher Scientific, BR0014) with 5% Tween-20 (Sigma, P1379) (PBS-T). Membranes were incubated with primary antibodies overnight at 4 °C with the following dilutions in 5% milk/PBS-T; anti-GAPDH (antibodies.com, A85271, 1:20,000), anti-PARP1 (Santa Cruz, sc-8007, 1:1000), anti-αPAR (Millipore, MABE1016, 1:1000), anti-MGMT (antibodies.com, A99874, 1:500), anti-nestin (Abcam, ab6142, 1:500), anti-SOX2 (Santa Cruz, sc-365823, 1:500), anti-p53 (Cambridge Bioscience, A300-247A, 1:1000), anti-BRCA1 (Santa Cruz, sc-6954, 1:200), anti-HSP70 (Santa Cruz, sc-32239, 1:1000) and CD133 (Abcam, ab316323, 1:1000) in 3% BSA/PBS-T. Membranes were washed 3× with PBS-T, each wash lasting 5 mins. Membranes were then incubated with secondary antibodies conjugated to HRP, all at 1:1000 in 5% milk with PBS-T for 1 h: anti-rabbit (DAKO, P0399) or anti-mouse (DAKO, P0447). Membranes were washed 3 times in PBS-T and protein bands visualised using Pierce ECL Western blotting substrate and developed using medical x-ray film and a Konica SRX 101 A Processor.

### 2.7. Olaparib/TMZ and Irradiation Treatments

PARP1 inhibitor Olaparib/Lynparza/AZD2281 (Adooq Bioscience, A10111; PARPi) and TMZ (Sigma Aldrich, T2577) was diluted with DMSO to make 10 mM stocks, which were stored at −20 °C. DMSO was added as a vehicular control to maintain consistent DMSO concentrations. DMSO, Olaparib and TMZ were diluted in GSC culture media to the final intended concentrations and 1 mL of the drug/DMSO dilutions was added to the desired wells. To firstly determine the inhibitory dose of Olaparib, 3D GSCs were incubated with Olaparib for 1 h followed by ionizing radiation (IR) or sham irradiated using a Caesium-137 (^137^Cs) Irradiator (CIS IBL437c) to a total dose of 5 Gy. SDS-PAGE analysis of subsequent lysates determined inhibition through loss of αPARylation (500 nM) in response to IR. For combination chemosensitisation studies, 3D GSCs were treated with DMSO or Olaparib (500 nM) and incubated for one hour before TMZ (5 mM) was added (where required) followed by exposure to TTFields (72 h, 200 kHz, 1.33 V/cm RMS), or incubated at 37 °C for the untreated ‘sham irradiated’ control. Cell survival was determined as detailed above. 

### 2.8. Statistical Analysis

Statistical significance was determined using the nonparametric one-way ANOVA comparing the indicated treatment to DMSO controls or to another indicated treatment cell population, and represented as follows: ns = not significant, * *p* < 0.05, ** *p* < 0.01, *** *p* < 0.001 and **** *p* < 0.0001.

## 3. Results

### 3.1. Monitoring of 3D GSC Cell Growth to Optimal TTFields Delivery Duration

**Figure 1:**
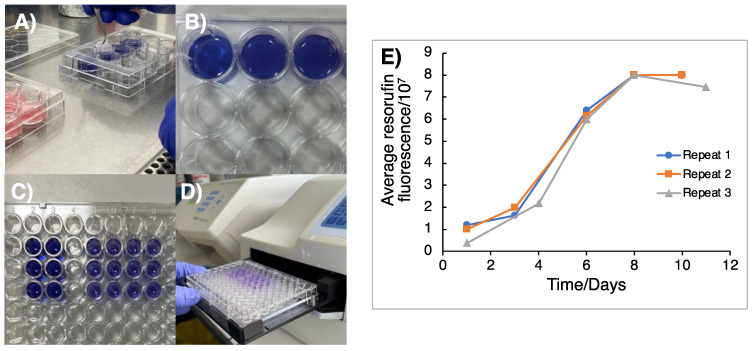
Colorimetric assay to determine cell doubling time of GSCs grown in 3D matrices. (**A**) Cells that were previously plated onto 3D Alvetex^TM^ scaffolds were moved from the 12-well dish into 2 mL of 1:20 alamar blue solution. (**B**) Scaffolds were then incubated in alamar blue solution at 37 °C for 1 h and then moved back into fresh media in a 12-well plate. (**C**) 200 μL of alamar blue solution was then transferred into a 96-well plate in duplicate. (**D**) Resorufin fluorescence was then measured using a plate reader to measure the metabolic activity of resazurin reduction. (**E**) Fluorescence of resorufin as a result of resazurin reduction was plotted against time in days to monitor cell doubling, with the cell doubling time being determined from the time taken for the fluorescence signal to double in the exponential growth phase of the curve, with an average taken over three independent repeats (*n* = 3). From this, the doubling time of G1 cells was calculated as 2.44 ± 0.33 days).

The cell doubling time of the GSC cultures was ascertained prior to the TTFields experiments in order to establish an appropriate duration for TTFields delivery to the 3D scaffolds. This is an important parameter to set given that one cell doubling time permits for each cell in a population to have progressed through at least one phase of mitosis and hence allows sufficient time for TTFields-mediated anti-mitotic effects to occur [17,20]. Cultured GSCs were firstly seeded onto Cultrex^®^-coated Alvetex^TM^ discs at a cell-line dependent density of 2 − 5 × 10^4^ cells per well and then left overnight to adhere and invade into the Alvetex^TM^ scaffold. Given that complete cell extraction and counting cannot be reliably performed in Alvetex^TM^-seeded GSCs, alamar blue colorimetric assays were used in substitution to monitor cell growth within the 3D Alvetex^TM^ scaffolds. Additionally, as alamar blue is a non-toxic metabolite, its use allows for the continued measurement of a specific well in a condition through the reduction of resazurin to resorufin, which is proportional to the cell number contained within each Alvetex^TM^ scaffold, and so the time-dependent increases in resorufin fluorescence can be used to measure cell proliferation (Figure 1). Alamar blue stock was firstly prepared by dissolving resazurin salt (0.3 mg/mL, TBS) before sterile filtration and protection from light for subsequent storage at 4 °C in between use. Alamar blue stock was diluted 1:20 in warmed GSC culture media, with 2 mL added to each well of a 12-well plastic dish. Using tweezers, Alvetex^TM^ discs were carefully moved and submerged into alamar blue solution and incubated (1 h, 37 °C) to allow for metabolic reduction of resazurin to resorufin (Figure 1A–E). After 1 h, 200 μL of GSC-exposed alamar blue solution was added to each well of a 96-well plate (in duplicate per condition) and measured by a fluorometric plate reader (resorufin: λ_ex_ 560 nm, λ_em_ 590 nm; Figure 1E). The Alvetex^TM^ scaffolds could then be returned to the original 12-well plate and resecured with the plastic insert. 

### 3.2. Appropriating 3D GSC Cultures for Use with the Inovitro^TM^ Preclinical TTFields System

**Figure 2:**
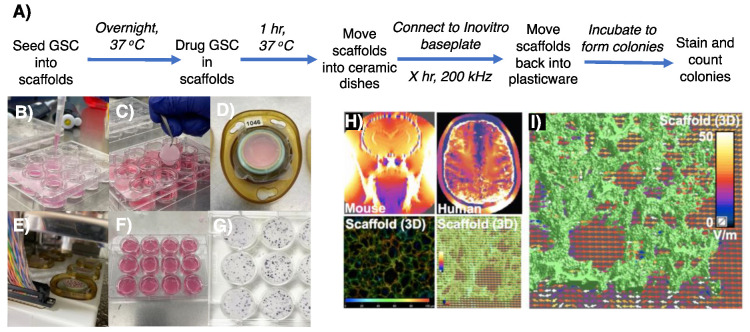
Procedure to assess preclinical TTFields regimens and simulation of TTFields delivery within 3D Alvetex^TM^ scaffolds. (**A**) A schematic which outlines a protocol to test TTFields alongside drug regimens on 3D Alvetex^TM^ GSC models. (**B**) Cells were seeded onto Cultrex^®^-coated Alvetex^TM^ scaffolds and incubated in 2 mL of stem media overnight. (**C**,**D**) With sterile tweezers, Alvetex^TM^ scaffolds were moved into the inovitro^TM^ ceramic dishes, secured in place with an insert and sealed with parafilm. (**E**) Ceramic inovitro^TM^ dishes were connected to the incubated base plate to deliver TTFields. (**F**) After the treatment time, scaffolds were returned back to their 12-well plastic plate, secured into place and incubated in fresh media to allow colony formation. (**G**) To visualise colonies, scaffolds were exposed to MTT solution (200 μL/condition, stock 10 mg/mL in PBS) and incubated for 4 h. When colonies were visible, media was aspirated, inserts removed and colonies counted to ascertain cell survival. (**H**,**I**) Computer modelling measuring the penetrative power of TTFields throughout 3D Alvetex^TM^ scaffolds, showing effective deliverance with non-uniform, more clinically relevant penetrative power, as seen in a mouse and human model.

The inovitro^TM^ system developed by Novocure is commonly used to deliver TTFields within 2D assays using glass-coverslip-plated cells [30], which we have recently used to improve the effectiveness of DDRi and radiotherapy-mediated cell killing of resistant GSCs [20]. The cell monolayers adherent to glass coverslips are usually treated with chemoradiotherapy before, during or after being moved into the inovitro^TM^ ceramic dishes and treated with TTFields, followed by cell detachment then replating to assess survival through clonogenic assays. To appropriate this methodology for 3D GSC cultures, cells were instead seeded into Alvetex^TM^ scaffolds and incubated to permit invasion and establishment within the micropores of the Alvetex^TM^ (Figure 2B). The next day, GSCs within the 3D matrices were then treated with chemotherapy compounds, radiation and/or DDRi prior to TTFields delivery. Following drug/radiation treatment, Alvetex^TM^ scaffolds were carefully moved with sterile tweezers into inovitro^TM^ ceramic dishes which contained 2 mL of GSC media, complemented with drug(s) if so required (Figure 2C,D). Ceramic inovitro^TM^ dishes were then connected in at least triplicate per condition in order to account for survival variation arising from TTFields delivery within the inovitro^TM^ system (Figure 2E). 

TTFields were then delivered with precise temperature control through voltage modulation with feedback from continuous temperature measurement within each dish, to achieve and maintain 37 °C dish temperature with 5% CO_2_ within an ESCO refrigerated incubator. Note: it is important to predetermine the exact temperature of the refrigerated incubator required as this determines the TTFields treatment intensity (V/cm RMS) delivered, as heating produced by TTFields is used to maintain each dish precisely at 37 °C [29]. After TTFields treatment, the scaffolds were moved with sterile tweezers back into the 12-well plastic dish and secured back into place with the insert in 2 mL of fresh GSC culture media and incubated at 37 °C until colony formation (Figure 2F,G). 

This provides a major technical advantage compared to 2D assays, as the 3D scaffolds are able to better accommodate the higher seeding densities required to generate meaningful survival data without significant colony overlap (Figure 2H,I). Therefore, the final seeding density is already preseeded into the scaffolds before TTFields delivery. Our approach offers the benefit of eliminating any additional variability and technical time associated with replating cells from each condition. Note: as GSC colony formation within the scaffolds cannot reliably be visualised using light microscopy, a predetermined colony formation time for each cell model should be conducted in order to establish the optimal duration of incubation for 3D colony formation. MTT reagent provides a suitable way of colony staining within the 3D matrices, whereby insoluble purple formazan salt is metabolically formed in living cells which stains colonies purple but does not stain the Alvetex^TM^ scaffolds (unlike crystal violet), aiding visual colony counting (Figure 2G).

**Figure 3:**
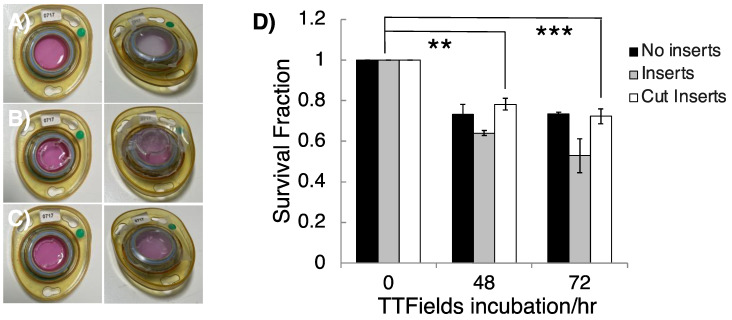
The effects of plastic inserts and TTFields incubation time on 3D GSC survival. Inovitro^TM^ ceramic dishes were constructed by moving a GSC cultured 3D Alvetex^TM^ scaffold with (**A**) no insert, (**B**) the provided plastic insert to create a domed parafilm seal or (**C**) cut inserts to create a flat parafilm seal. (**D**) G1 GSCs were cultured and subjected to TTFields (200 kHz, 1.33 Vcm RMS) for 48 and 72 h in ceramic inovitro^TM^ dishes with no inserts, cut inserts and inserts, and their clonogenic survival was measured after colony formation. *n* = 3. One-way ANOVA statistical test indicated significant cell killing; 0 h vs. 48 h for cut inserts (*p* = 0.0026 **; 0 h vs. 72 h (cut inserts), *p* = 0.0007 ***).

Having established a method to evaluate TTFields treatment regimens using 3D GSC clonogenic survival, further optimisation was carried out in order to create a robust assay that minimised media evaporation due to the presence of the plastic inserts that are required to prevent floatation of the Alvetex^TM^ matrices, which is observed when scaffolds are moved into ceramic dishes without them. This was explored by testing the effects of the plastic inserts by connecting ceramic dishes composed of scaffolds with no insert, scaffolds with the provided insert and scaffolds with modified inserts, which were cut down to match the height of the ceramic dishes. In addition, GSC cell survival within a TTFields incubation time, that is within a time interval less than one cell doubling time, was also assessed (Figure 3) and was comparable to the TTFields-induced toxicity that we have recently reported for the same GSC model in 2D cultures [20].

Cell survival was not significantly altered between 48 and 72 h of 200 kHz, 1.33 Vcm RMS TTFields (G1 cell doubling time is 2.44 ± 0.33 days), consistent with a large component of the therapeutic impact of TTFields therapy being attributable to anti-mitotic effects [17]. Despite small, measured survival differences depending on the insert types, dishes with either cut or no inserts were the most compatible with the inovitro^TM^ system due to a flat parafilm seal which minimised media evaporation avoiding any requirement for media top up during treatment. In contrast, ‘doming’ of the parafilm seals in dishes that contained full height inserts led to persistent registered errors on the inovitro^TM^ system and application difficulties arose due to extensive media evaporation, which caused disconnection of the ceramic dishes, leading to higher background toxicity. We therefore conclude that the use of modified (cut) Alvetex^TM^ inserts, as described, makes the use of such 3D matrices compatible with the inovitro^TM^ TTFields system. 

### 3.3. Optimisation of Cell Lysis Following 3D TTFields Delivery for Molecular Analyses

**Figure 4:**
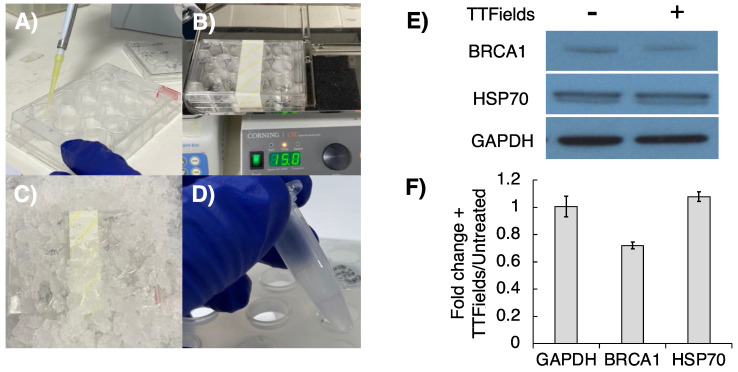
Examining protein expression following TTFields delivery to 3D Alvetex^TM^ cultured GSCs. Following TTFields treatment (72 h, 200 kHz, 1.33 Vcm RMS). (**A**) Scaffolds were moved back into a 12-well plastic dish and lysed with lysis buffer (100 μL/well). (**B**) Lysis was promoted by agitation on a plate shaker (150 rpm, 15 min) and then (**C**) placed on ice for 15 min. (**D**) Lysates were transferred to Eppendorfs, kept on ice for 30 min and centrifuged, and the supernatant was collected. (**E**) Western blot analysis examined the effects of TTFields on BRCA1 expression (Figure S1). (**F**) Band densiometry analysis showed hyperthermia-independent depletion of the BRCA1 protein in response to TTFields (mean −/+ SEM; *n* = 3).

Following optimisation of efficient/robust TTFields delivery in 3D GSC cultures, we next developed a similar robust protocol for protein extraction from these 3D TTFields-treated cultures, so that key post-translational regulatory effects on the DNA damage response (DDR) pathways could be monitored, as we have done recently within 2D cultures [20]. GSCs were seeded into Alvetex^TM^ scaffolds (12-well plate format) at a density of ~15 × 10^4^ cells/well and incubated overnight. TTFields delivery was then conducted as described above. After TTFields delivery, the Alvetex^TM^ scaffolds were moved back into a 12-well plastic dish and washed with cold PBS (2 × 1 mL/well) before the addition of 100 μL/well of lysis buffer (see above). Plates were placed on ice for 10 min before being placed on a shaking platform (150 rpm, 15 min) to permit complete cell lysis throughout the scaffold. After this, the plates were tilted and lysates collected into 0.5 mL Eppendorf tubes and placed on ice for 30 min, with vortexing every 15 min before centrifugation (13,000× *g*, 4 °C, 15 min). The supernatant containing extracted protein was then collected, with the protein concentration measured using a relevant Bradford-type assay (Figure 4A–D), before subsequent SDS-PAGE/Western blot analysis. Encouragingly, G1 cells subjected to TTFields (72 h, 200 kHz, 1.33 Vcm RMS) using this methodology were found to exhibit reduced BRCA1 expression in comparison to the TTFields-untreated GSC control (Figure 4E,F), which is consistent with previous findings in 2D TTFields cancer cell models [31,32,33].

### 3.4. TMZ and Olaparib Enhance TTFields-Mediated Cell Toxicity within 3D GSC Models of Spatial Heterogeneity and Residual Disease

TMZ is a DNA methylating agent that is used worldwide as the standard-of-care chemotherapeutic against newly diagnosed glioblastoma, including to treat residual invasive-edge cancer cells left behind following surgical resection [34]. More recently, the PARP1 inhibitor (PARPi) Olaparib/Lynparza has been incorporated into treatment regimens for breast, prostate and ovarian cancer, including strategies delivering chemoradiosensitisation of BRCA-deficient tumours, and is currently undergoing clinical trial assessment for glioblastoma [35,36,37,38]. The use of TMZ and PARPi in cancer treatment therefore provides a strong preclinical rationale to test their combined efficacy with TTFields-mediated downregulation of FA/BRCA proteins [31,32,33]. 

**Figure 5:**
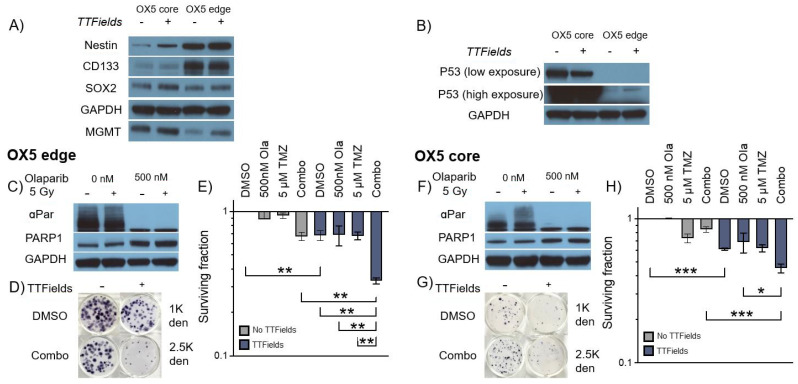
TTFields alongside Olaparib (PARPi) enhances 3D GSC cell death by TMZ. (**A**) Western blot analysis validating MGMT expression, GSC phenotype and intratumoural heterogeneity of 3D grown OX5 core and edge GSCs through the expression of previously validated GSC markers (Nestin, CD133 and SOX2) with and without TTFields incubation (200 kHz, 1.33 Vcm RMS 72 h). (**B**) Western blot analysis validating the differences in p53 expression between 3D-grown OX5 core (mutated) and OX5 edge cells (wildtype) with and without TTFields incubation (200 kHz, 1.33 Vcm RMS 72 h). (**C**,**F**) Western blot analysis validating Olaparib (500 nM) PARP1 inhibition in 3D GSCs through depletion of alpha-Par signal in response to radiation (5 Gy) in OX5 edge and OX5 core cells, which respectively represent GSCs derived from the resected tumour core and residual invasive margin. (**D**,**G**) Representative images of 3D colonies in the indicated treated OX5 core and edge models at the indicated cell density. (**E**,**H**) Clonogenic survival assays of cells pretreated with Olaparib (500 nM, 1 h) and TMZ (5 μM) before TTFields incubation (200 kHz, 1.33 Vcm RMS 72 h) in OX5 edge (**C**) and OX5 core (**F**) cells. Statistical significance is denoted through ANOVA one-way analysis (*n* = 3), * *p* < 0.05, ** *p* < 0.01 and *** *p* < 0.001.

We therefore used our optimised 3D GSC TTFields delivery protocol in a matched MGMT+ (a genetic/cellular marker which confers a poorer clinical TMZ response [1]) glioblastoma tumour core (typically resected) and edge (typically residual) model [27,28] to test these multi-modal regimens and provide proof-of-concept data around our preclinical methodology (Figure 5). Additionally, using these tumour core/edge models’ therapeutic responses to reflect treatment-naïve disease from the typically contrast-enhancing tumour bulk (core), potentially supporting efficacy data for regimens to treat patients where surgical resection is not feasible, as well as in distant brain-invasive margin GSCs (edge), which are more reflective of typical post-surgical residual disease following current standard-of-care treatment, can be assessed. MGMT expression was validated in both OX5 core and edge cell models alongside the presentation of the GSC phenotype through the historically established GSC markers Nestin, CD133 and SOX2 (Figure 5A), confirming both the TMZ resistance and intratumoural heterogeneity of the model [27,28]. The expression of these markers persisted despite TTFields treatment, which shows a phenotypic GSC retention throughout the treatment regimen.

Encouragingly, TTFields effectively reduced GSC survival in combination with TMZ and Olaparib at concentrations validated to be deliverable to the brain [38,39]. This data therefore highlights the utility of our 3D preclinical TTFields delivery methodology to facilitate the assessment of a range of therapeutic TTFields combinations in clinically relevant primary 3D GSC models. Interestingly, the p53 status of OX5 core and edge cells differed (Figure 5B), with OX5 core cells exhibiting hyper expression of a common inactivated p53 form (Arg273 > His), whilst OX5 edge remained p53 WT (confirmed by WES analysis; data not shown). Regardless of this, both OX5 core and edge GSCs were sensitized to TMZ by TTFields and potentiated Olaparib treatments, thus highlighting how our preclinical 3D model may be used to identify potential TTFields-based strategies to potentially overcome common treatment resistive barriers.

## 4. Discussion

Given the approval of TTFields therapy for the treatment of both primary and recurrent glioblastoma in the USA as well as several countries across Europe and Asia, and the current interest in developing DDR-targeting therapies to enhance current SoC TMZ/IR therapies including TTFields [11,17,20], we set about developing and optimising a robust medium to high throughput 3D GSC-based preclinical TTFields application methodology to facilitate translation preclinical studies. The 3D Alvetex^TM^ cell culture system has been previously validated to induce cytoskeletal rearrangements and effect EGFR/VEGFR presentation, more similar to in vivo tumours, and therefore it can predict responses to EGFR/VEGFR-based therapies and radiotherapy more accurately [23,24], highlighting the benefits of 3D-based culture models; however, no previous studies have reported use of the 3D Alvetex^TM^ system with TTFields. Here, we provide an optimised and robust methodology to facilitate preclinical 3D-based TTFields studies that can incorporate the assessment of TTFields on primary spatio-heterogeneity GSC sub-populations, either alone or alongside existing or experimental chemoradiotherapy treatments.

Other TTFields-appropriated 3D models exist which have been used to test treatment responses using rodent-grown, patient-derived orthotopic slices and/or organoids [40,41,42]. Such 3D models present multiple advantages when compared to 2D cultures, such as mimicking 3D brain architecture, the establishment of complex cell–cell interactions and the possession of ECM components, and they can therefore reliably measure treatment responses. However, the increase in the complexity of these models portends an increase in labour and time, a reduction in throughput capacity, additional culturing steps and challenges to reproducibly generating and efficiently interpreting efficacy data. In comparison, Alvetex^TM^ scaffolds offer a faster and simpler culturing methodology, resulting in increased throughput and a more robust testing pipeline at the preclinical stage. Additionally, organoids and/or orthotopic slice cultures contain a less predictable mixture of cells, with various degrees of stemness or differentiation, in part due to limited media penetration into the structures, whereas 3D GSCs within Alvetex^TM^ scaffolds are enriched to present the most treatment-resistant phenotypes, which can therefore be used to predict how effective a regimen is against resistant cells which are most likely to underpin residual-to-recurrent tumour regrowth [43,44]. 

Furthermore, molecular analysis of subsequent effects in organoid/orthotopic slices are often more complex and time inefficient than with 3D-scaffold-based GSCs. Additionally, the ease and simplicity of the 3D model presented here compared to our recently reported 2D-based TTFields approaches [20], such as its elimination of the need for cells to be counted and replated post treatment, facilitates a higher throughput methodology. Finally, akin to 2D methods, 3D cultures grown within the Alvetex^TM^ scaffolds can be lysed directly for protein/RNA/DNA extraction to ease downstream proteomic, transcriptomic and genetic analyses.

## 5. Conclusions

Historically, 2D cell culturing presents the most common model for testing TTFields-based effects as part of preclinical studies. However, previously reported flaws in the inability of 2D culturing to accurately predict clinical responses of gliomas to chemoradiotherapy provides a rationale to develop a robust, adaptable 3D platform for TTFields-based preclinical glioma studies. Furthermore, extensive sub-cellular niches within glioblastomas promote and maintain the treatment-resistant GSC phenotype, representing a second barrier of heterogeneity which is lost in 2D cell culture. We therefore believe that the 3D TTFields methodology presented here, which we show is compatible with parallel patient-derived GSC models reflecting intra-tumoural spatial heterogeneity, a known driver of therapy resistance, will act as a platform to facilitate future preclinical assessment of potentially impactful therapeutic regimens involving TTFields-based approaches to help identify much-needed novel treatment approaches for these currently incurable tumours.

## Data Availability

The data presented in this study and that support these studies are available on request from the corresponding authors. Note that not all data can currently be made publicly available due to ethical considerations around patient material.

## Supplementary Materials

The following supporting information can be downloaded at: https://www.mdpi.com/article/10.3390/cancers16050863/s1, Figure S1: Original blots/densitometry.
