# Supplementary material for: Development and Optimisation of Tumour Treating Fields (TTFields) Delivery within 3D Primary Glioma Stem Cell-like Models of Spatial Heterogeneity
Source: Cancers (Basel). 2024 Feb 21;16(5):863. doi: 10.3390/cancers16050863 (PMC10930697; doi:10.3390/cancers16050863)
Supplement: Supplementary file 1 [file cancers-16-00863-s001.zip › Figure S1.pdf]

Original blots/densitometry (Jones *et al*)

Figure 4E

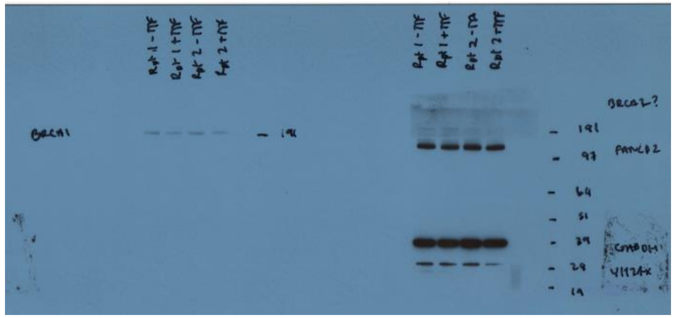

Figure 4E

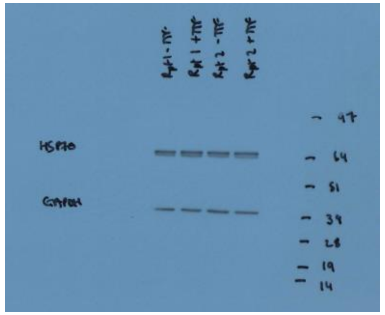

Figure 4F

Protein	Condition/Lane	Densiometry area
GAPDH	G1 Rpt 1 - TTF	19702.765
	G1 Rpt 1 + TTF	21307.522
	G1 Rpt 2 - TTF	24516.3
	G1 Rpt2 + TTF	22832.815
HSP70	G1 Rpt1 - TTF	27408.999
	G1 Rpt1 + TTF	30516.978
	G1 Rpt2 - TTF	27787.099
	G1 Rpt2 + TTF	29009.513
BRCA1	G1 Rpt1 - TTF	21155.563
	G1 Rpt 1 + TTF	15763.25
	G1 Rpt2 - TTF	25052.827
	G1 Rpt2 + TTF	17406.371

Figure 5A and 5B

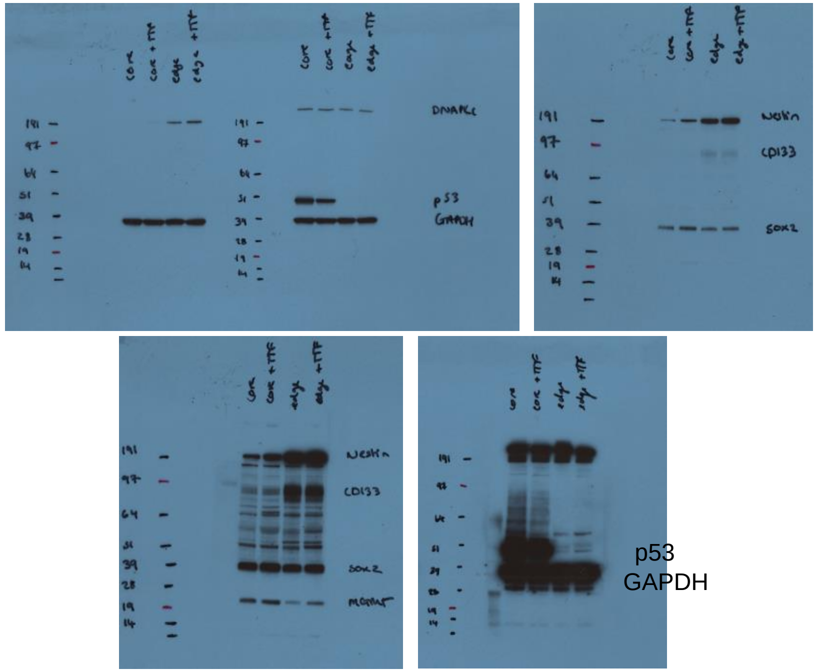

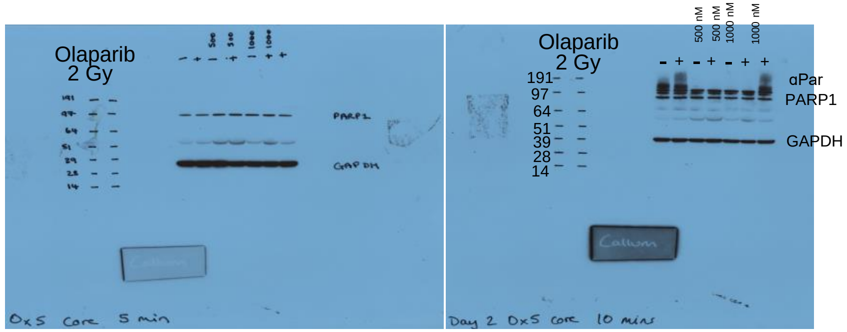

Figure 5C

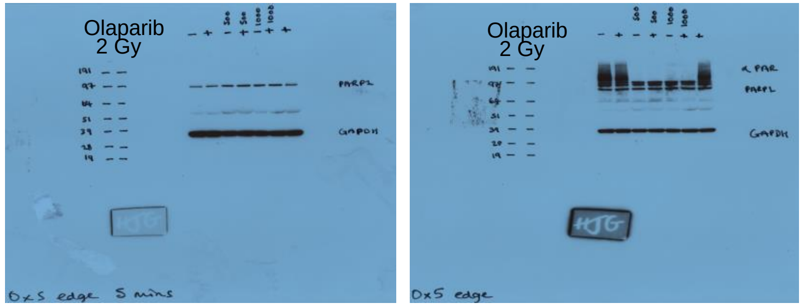

Figure 5F
